# BASE: A novel workflow to integrate nonubiquitous genes in comparative genomics analyses for selection

**DOI:** 10.1002/ece3.7959

**Published:** 2021-09-03

**Authors:** Giobbe Forni, Angelo Alberto Ruggieri, Giovanni Piccinini, Andrea Luchetti

**Affiliations:** ^1^ BiGeA Department University of Bologna Bologna Italy; ^2^ Department of Biology University of Puerto Rico–Rio Piedras San Juan Puerto Rico

**Keywords:** CodeML, d*N*/d*S*, molecular evolution, omega, selective pressures

## Abstract

Inferring the selective forces that orthologous genes underwent across different lineages can help us understand the evolutionary processes that have shaped their extant diversity and the phenotypes they underlie. The most widespread metric to estimate the selection regimes of coding genes—across sites and phylogenies—is the ratio of nonsynonymous to synonymous substitutions (d*N*/d*S*, also known as *ω*). Nowadays, modern sequencing technologies and the large amount of already available sequence data allow the retrieval of thousands of orthologous genes across large numbers of species. Nonetheless, the tools available to explore selection regimes are not designed to automatically process all genes, and their practical usage is often restricted to the single‐copy ones which are found across all species considered (i.e., ubiquitous genes). This approach limits the scale of the analysis to a fraction of single‐copy genes, which can be as low as an order of magnitude in respect to those which are not consistently found in all species considered (i.e., nonubiquitous genes). Here, we present a workflow named BASE that—leveraging the CodeML framework—eases the inference and interpretation of gene selection regimes in the context of comparative genomics. Although a number of bioinformatics tools have already been developed to facilitate this kind of analyses, BASE is the first to be specifically designed to allow the integration of nonubiquitous genes in a straightforward and reproducible manner. The workflow—along with all relevant documentation—is available at github.com/for‐giobbe/BASE.

## Graphical abstract

Comparative genomics analyses for selection are often restricted to the subset of genes which is shared by all the species considered (i.e., ubiquitous genes) while those which are not found across some of them (i.e., nonubiquitous genes) are often overlooked, due to the lack of automated approaches to include them. Yet, disregarding such a large portion of genes may potentially conceal important evolutionary processes. For this reason, we developed a novel workflow—named BASE—which allows the integration of nonubiquitous genes in a straightforward and reproducible manner.

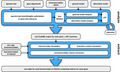

## 1 INTRODUCTION

Selection can shape the evolution of protein‐coding genes by preventing changes in their sequences (purifying selection) or through the fixation of novel adaptive variants (positive selection). Quantifying its nature and strength is a key step to understand the diverse evolutionary histories of orthologous genes across different species and clades. Statistical models of molecular evolution have proven to be fundamental approaches to investigate such processes and can be divided into those based on comparing divergence and segregating polymorphism—such as the MK test and its extensions (McDonald & Kreitman, 1991)—and those based on multi‐species sequence divergence—also known as codon models. The two approaches use different conceptual frameworks and are better applied for analyses at different timescales, with the first approach more suited to investigate recent processes and the latter ones more apt to infer older events (Mugal et al., 2014).

Approaches based on sequence divergence among multiple species are cornerstones in the estimations of patterns of sequence evolution and selection regimes. After the first models were developed to infer the strength of selection on coding sequences globally across their sites and species phylogeny (Goldman & Yang, 1994; Muse & Gaut, 1994), subsequent elaborations allowed for variation across lineages (Yang, 1998), sites (Anisimova et al., 2001; Nielsen & Yang, 1998; Yang et al., 2000), and both (Yang & Nielsen, 2002; Zhang et al., 2005). Pairwise comparisons between models can be performed using likelihood‐ratio tests (LRTs) to understand which one better reflects the molecular evolution of a group of orthologous genes (Anisimova et al., 2001). The interpretation of all these models is largely based on the d*N*/d*S* parameter (Kimura, 1977; also known as *ω*), which is calculated as the ratio of nonsynonymous substitution rates (nonsynonymous mutations over nonsynonymous sites; d*N*) to synonymous substitution rates (synonymous mutations over synonymous sites; d*S*). This metric is fundamental to investigate the extent to which selection modulates sequence evolution of the protein‐coding portions of genes. While d*S* are assumed to evolve neutrally, d*N* are expected to be exposed to selection, as they change the amino acid structure of proteins. Despite the fact that some of these assumption have been challenged (Davydov et al., 2019; He et al., 2020; Kryazhimskiy & Plotkin, 2008), analyses based on codon models have proved themselves as key approaches in comparative genomics, such as investigating positive selection connected to evolutionary innovations (Li et al., 2014; Parker et al., 2013; Zhang et al., 2014) or testing the relaxation of selective constraints after trait decay (Liu et al., 2019; Policarpo et al., 2020). In other instances, these approaches have been used to observe genome‐wide effects linked to events such as shifts in environmental niches or the loss of recombination in asexual species (Bast et al., 2018; Plazzi et al., 2017).

Several pieces of software have been developed to infer codon models for coding sequences: Selecton (Stern et al., 2007), HyPhy (Pond et al., 2005), TreeSAAP (Woolley et al., 2003), and the CodeML program in the PAML package (Yang, 2007). The latter program was also subject to several implementations, such as IDEA (Egan et al., 2008), PAMLX (Xu & Yang, 2013), SlimCodeML (Valle et al., 2014), IMPACT_S (Maldonado et al., 2014), LMAP (Maldonado et al., 2016), ete‐evol in the ete3 package (Huerta‐Cepas et al., 2016), VESPA (Webb et al., 2017), BlastPhyMe (Schott et al., 2019), and EasyCodeML (Gao et al., 2019). With the increment of genomics and transcriptomics studies, it has become rather common to infer selective regimes of thousands of genes for hundreds of species and all of aforementioned CodeML implementations mainly try to overcome its limited ease of use in the context of comparative genomics.

**FIGURE 1:**
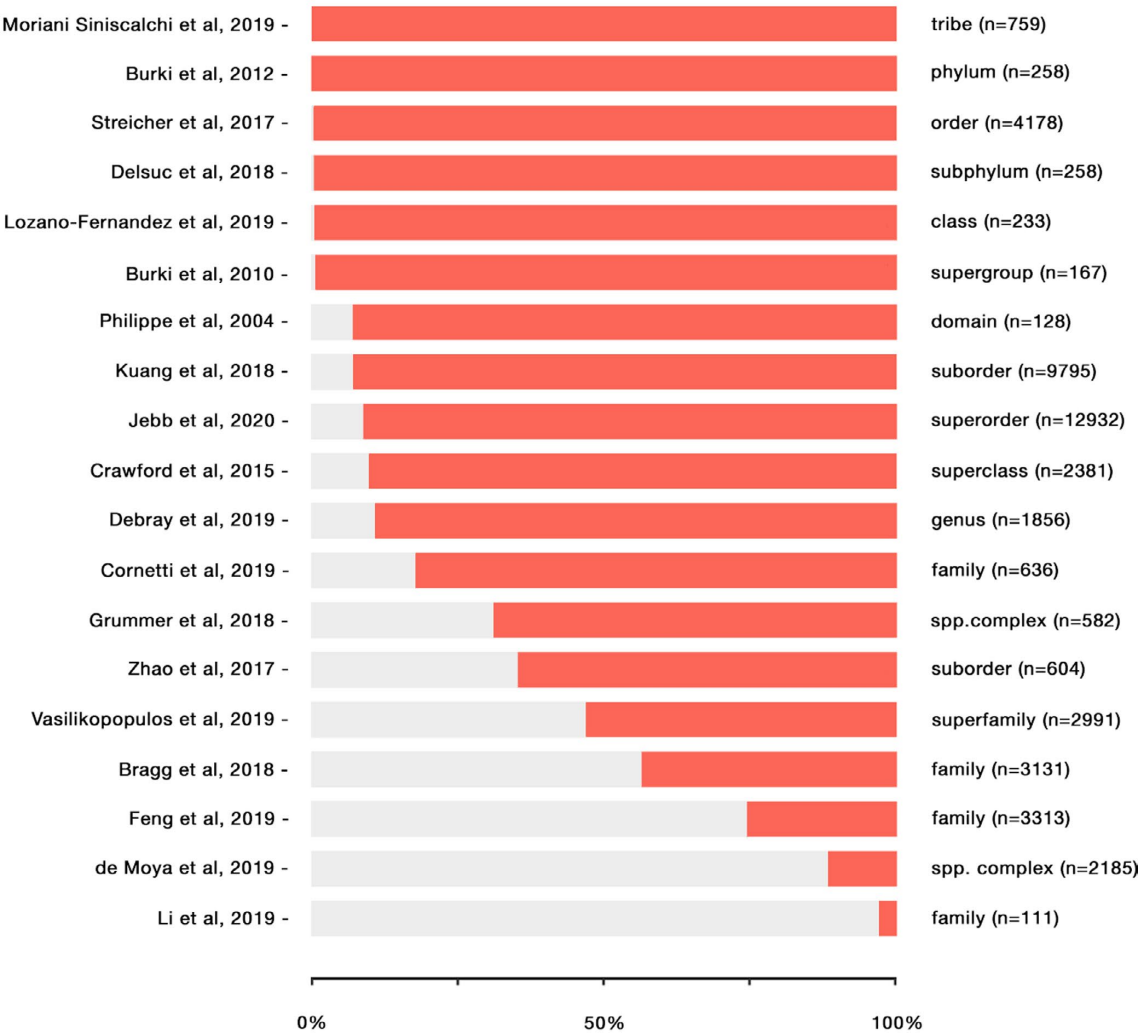
Comparative genomics datasets consistently include a large number of nonubiquitous genes. The proportion of ubiquitous and nonubiquitous genes was calculated in 18 published datasets, varying in their total number of genes and taxonomic level (from family to phylum). In our analysis, the average percentage of nonubiquitous genes is 73.4% while for ubiquitous ones is 26.6%

Our focus on developing BASE has been mainly directed to facilitate the integration of an often overlooked—yet incredibly large—portion of genomes in comparative genomics analyses for selection. Genes can differ in many aspects—such as being single‐copy or multi‐copy—and they can also be either shared by all species considered (i.e., ubiquitous genes) or not found in some of them (i.e., nonubiquitous genes). The latter case can be either due to biological or technical causes, but nonetheless a large number of single‐copy genes in comparative genomics datasets consist of nonubiquitous genes. As an exploratory example, we retrieved the proportion of ubiquitous and nonubiquitous genes from 18 published datasets, which have been generated for comparative genomic or phylogenomic purposes. While we tried to consider datasets varying in total gene number and taxonomic level, this overview has to be considered far from comprehensive: The outcome of orthology inferences is rarely included in publications, and thus, we largely relied on authors’ personal communications. Despite the partial nature of this analysis, it shows how comparative genomics datasets consistently include a large portion of nonubiquitous genes (Figure 1). The latter are mostly overlooked in selection analyses—which are typically based only on single copy and ubiquitous genes—due to the lack of automated approaches for their inclusion. Yet, disregarding such a large portion of genes may potentially conceal important evolutionary processes and for this reason we developed a novel workflow intended specifically for this purpose.

## 2 IMPLEMENTATION

**FIGURE 2:**
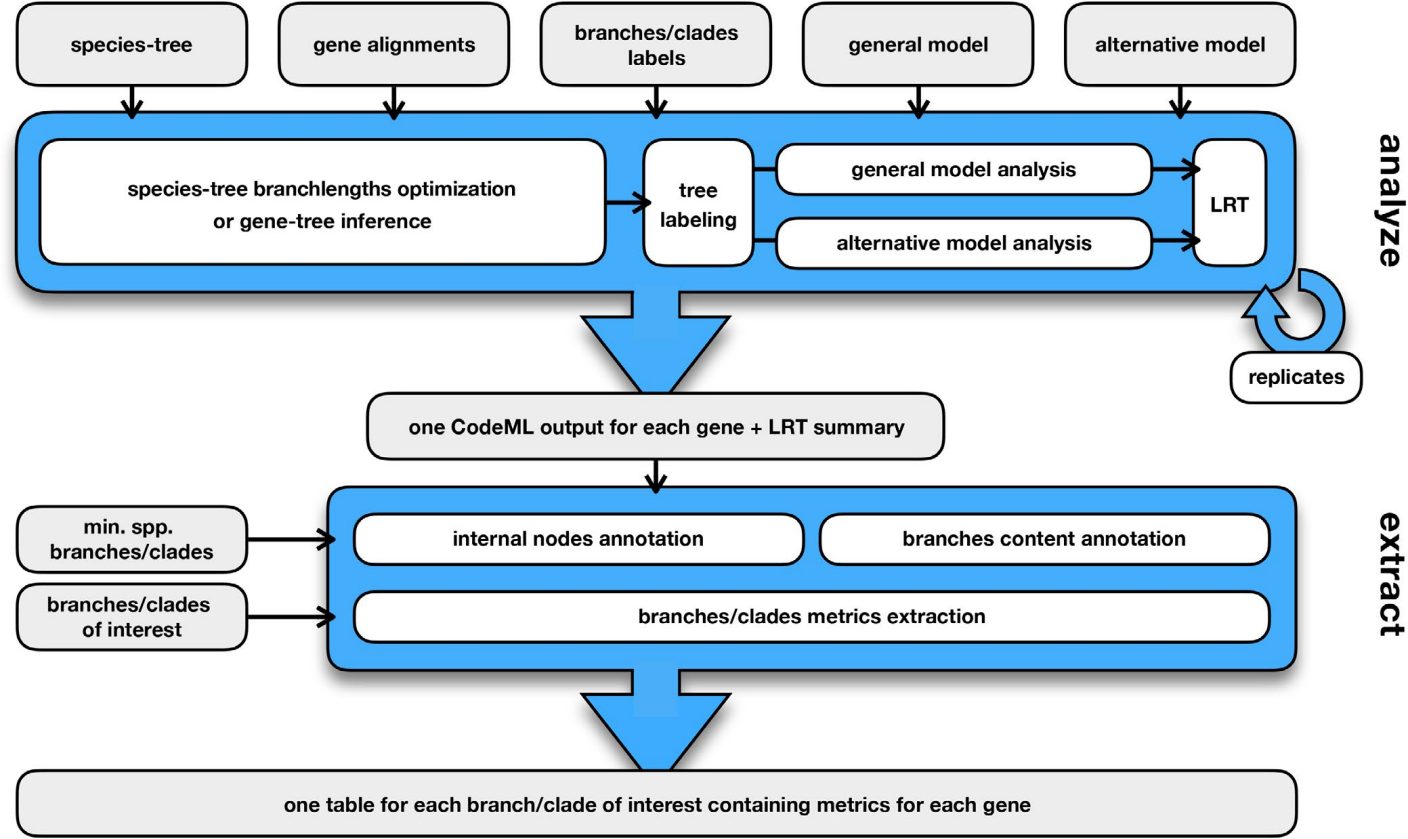
BASE workflow consists of the two steps "analyze" and "extract", which can be carried out independently. The "analyze" step requires a folder containing protein‐coding gene alignments (in fasta format) along with general and alternative nested models (two CodeML control files). The user can carry out the analysis leveraging a fixed species tree (in newick format) or inferring gene trees for each gene. Branch and/or clade labels can also be used, and all the complex schemes implemented in CodeML can be reproduced in BASE. Users can specify a number of replicate analyses, to avoid local optima in parameter‐rich analyses. The "extract" step requires CodeML outputs as inputs, including also those generated outside of the workflow; this step will retrieve metrics (d*N*d*S*, d*N*, and d*S*) relative to the branches and/or clades of interest. When nonubiquitous genes are included in the analyses, it is possible to include only genes which lack just species external to the branches/clades of interest or to include also genes which are not found in some species within it. In the latter case, BASE implements a threshold for missing species within the branches/clades of interest

BASE workflow is written in BASH and R and has been tested on Linux operating systems, such as centOS 8. As it extensively leverages GNU utilities, its usage is restricted to Linux distributions. It consists of two main steps: In the first one (“analyze”), evolutionary model parameters are inferred across alignment sites and tree branches for the different genes, while the subsequent step (“extract”) allows to retrieve the different metrics associated with specific branches or clades in the species tree. CodeML provides in large part the statistical and computational framework to perform these analyses and is at the core of the workflow, whose general description is reported in Figure 2.

### 2.1 “Analyze” step

The inputs required for the “analyze” step are (a) a folder containing protein‐coding genes alignments in fasta format; (b) two CodeML control files describing two nested models—where one is a specific case of the other; all the parameters in control files can be customized, supporting branch, site, and branch‐site models; moreover, other optional files can be required depending on analysis specifications: (c) a species tree in newick format, which can be multifurcating but has to include all the species present across the different gene alignments; and (d) a labeling scheme when the user wants to analyze models that assume specific clades and/or branch rates. The workflow initially checks the alignments for the presence of stop codons using Transeq of the EMBOSS package (Rice et al., 2000), excluding from subsequent analysis genes that include any. Either branch length optimization on the species‐tree topology or gene‐tree inference can be then carried out for each gene alignment, using RAxML with a codon‐aware GTR substitutions model (Stamatakis, 2014). Subsequently, the two CodeML analyses configured with the general and the alternative models are performed and compared through a LRT using R (R Core Team, 2013). Replicate analyses can be specified, so that the general and alternative model inferences will be carried out a user‐defined number of times and those with the "best" likelihood value will be used for the LRT. The resulting *p* values are automatically adjusted using a false‐discovery‐rate (FDR) correction; then, the codeml output relative to the best‐fit model is selected for each gene, and LRT outcome is summarized in a table. When leveraging models which assume specific clades and/or branch rates, the user can specify labels in a custom input file and BASE will automatically use them to annotate the trees using phangorn R package (Schliep, 2011). All the complex labeling schemes possible in CodeML can be replicated in BASE, which allows the use of multiple branch (#) and clade ($) labels in the same analysis. Moreover, also the inference and comparison of two models including labels are supported, as long as the alternative one is nested within the general one. Additional resources on labeling strategies are available in online tutorials. The default behavior of BASE is to process all genes, whether ubiquitous or nonubiquitous, but the user can limit the analysis to just ubiquitous ones. When the analysis is configured to consider also nonubiquitous genes, the species tree will be pruned on the basis of the species present using ape R package (Paradis et al., 2004), prior to species‐tree branch length optimization or gene‐tree inference.

### 2.2 “Extract” step

**FIGURE 3:**
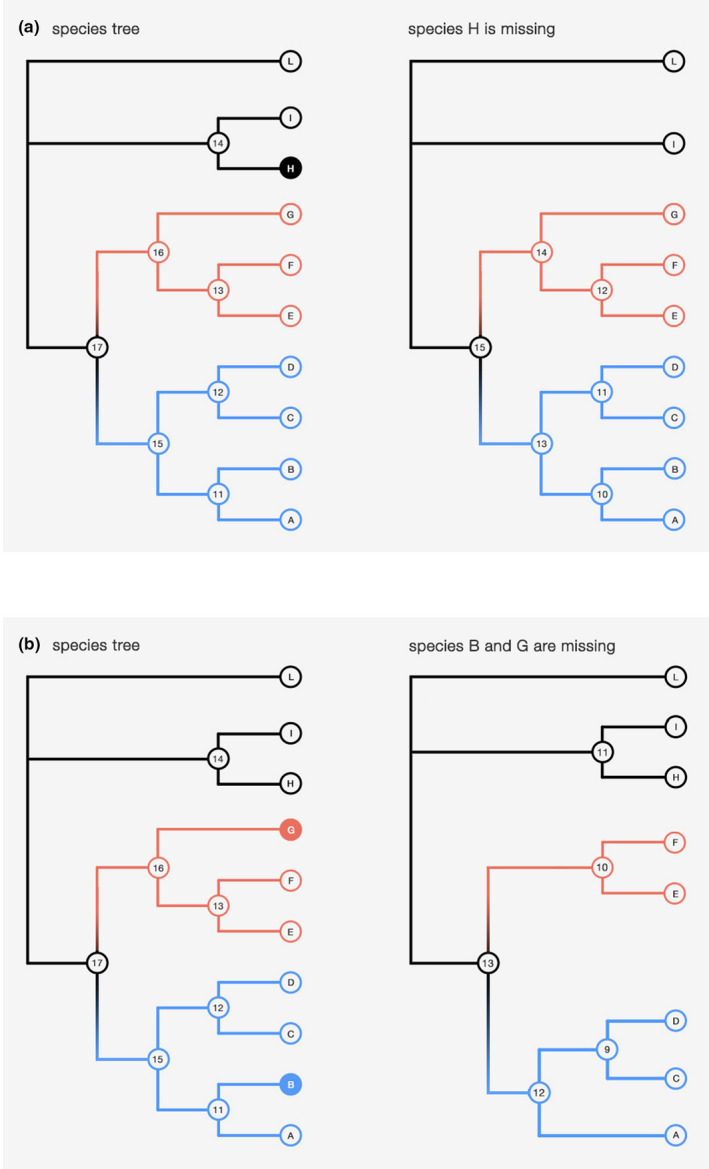
In this example, two clades of interest are highlighted in red and blue, but the same applies when specific branches are considered. Other than restricting the analysis to ubiquitous genes, BASE can include the following: (a; top panel) nonubiquitous genes which are not found only in species external to the clades of interest and (b; bottom panel) nonubiquitous genes which are not found also in species of the clades of interest. In this latter case, it is possible to implement a threshold for missing species within the clades/branches of interest. This example describes an analysis where a fixed species‐tree topology is used, but BASE allows also to leverage gene‐tree topologies

The “extract” step can be carried out subsequently to the “analyze” one, using as input: (a) the output folder generated by the previous step or a folder containing CodeML outputs generated by means other than BASE; and (b) a list of all branches and/or clades of interest, defined by their associated species. In the first place, this step will annotate internal nodes of each tree to match the output of CodeML and will list all species associated with each branch of the phylogeny. Subsequently, the pipeline will create a table for each branch/clade specified by the user, containing the d*N*/d*S*, d*N*, and d*S* values relative to the best‐fit model for each gene. Equivalent branches/clades can be identified in a phylogeny even in the absence of some species. For example, a clade—and its stem branch—made up of tens of species can be considered to be still present if we subtract a few species from the phylogeny, in either the in‐group or the out‐group. As such, in BASE "extract" step it is possible to include nonubiquitous genes with two approaches (Figure 3): (A) restrict analyses to genes which are present in all species of the branches/clades of interest but may not be found in external species or (B) also implement a threshold for missing species within the branches/clades of interest. This threshold can be specified by either an absolute number or a proportion (e.g., if 0.8 is specified, at least the 80% of each branch/clade species need to be present in a given gene in order to include it in the output). If a given branch or clade do not meet the selected criteria, this will be stated in the final output and no associated metrics will be reported. The inclusion of nonubiquitous genes should be applied with caution by the user: Too many missing species—both in the branches/clades of interest and/or in the whole tree—could impact evolutionary rates of inference. We suggest to either opt for (A) or to rely on conservative thresholds when opting for (B) (e.g., 90% of species present; van Kruistum et al., 2021).

### 2.3 Additional implementations

Even if the focal feature is the integration into selection analyses of single‐copy genes which are not found across all considered species, BASE also includes two other major technical implementations. The first one concerns analyses with a high number of parameters and which may encounter local likelihood optima: A good—yet often overlooked—practice is to run analyses multiple times with varying starting values in order to obtain the global optimum. BASE allows to seamlessly carry out a user‐specified number of replicate analyses, incorporating random omega starting values: The replicate which has the "best" likelihood value will be then used for further analyses. A second issue concerning phylogeny‐based selection analyses is the possibility of discordance between gene trees and species tree. This circumstance can underlie a wide range of technical and biological phenomena—such as sequence misalignment, nonorthology, and incomplete lineage sorting—which can ultimately bias evolutionary rate inference. In order to account for such possibility, when a fixed species tree is specified BASE will report its normalized Robinson–Foulds distances with each gene tree, calculated using ete3 (Huerta‐Cepas et al., 2016). The user can then decide to exclude analyses where there is a strong conflict between the gene‐tree and species‐tree topologies or to re‐launch them using the gene tree. This latter possibility can for example be leveraged to account for the artefactual substitution rate variation which occurs when substitutions on discordant gene trees are analyzed in the context of a fixed species tree (Mendes & Hahn, 2016). BASE also provides additional features that ease the inference, comparison, and interpretation of analyses on selection regimes, such as the automatic labeling and/or metrics retrieval for specific branches/clades in large phylogenies, simultaneous batch processing of genes to cut down processing times, and a large number of error messages which can definitely ease the user experience.

## 3 CONCLUSIONS

BASE is a workflow for analyses on selection regimes that integrates several popular pieces of software, with CodeML at its core. It has been conceived to allow the integration of nonubiquitous genes into comparative genomics analyses for selection in a straightforward and reproducible manner, yet it also implements many other features and quality‐of‐life improvements. We hope that BASE proves to be a useful tool for comparative genomics and that it generates some interest toward the frequent exclusion of such a vast portion of genes in selection analyses. BASE is an ongoing project, and we welcome bug reports, feedback, and suggestions for feature implementations. All the documentation, including detailed tutorials to explore BASE functionality, can be found at github.com/for‐giobbe/BASE.

## CONFLICT OF INTERESTS

Authors declare that they have no competing interests.

## AUTHOR CONTRIBUTIONS

**Giobbe Forni:** Conceptualization (lead); Investigation (lead); Methodology (lead); Software (lead); Writing‐original draft (lead); Writing‐review & editing (lead). **Angelo Alberto Ruggeri:** Conceptualization (supporting); Investigation (supporting); Methodology (supporting); Software (supporting); Validation (supporting); Writing‐original draft (supporting); Writing‐review & editing (supporting). **Giovanni Piccinini:** Conceptualization (supporting); Investigation (supporting); Methodology (supporting); Software (supporting); Validation (supporting); Writing‐original draft (supporting); Writing‐review & editing (supporting). **Andrea Luchetti:** Conceptualization (supporting); Methodology (supporting); Supervision (lead); Writing‐review & editing (supporting).

### OPEN RESEARCH BADGES

This article has earned an Open Data Badge for making publicly available the data necessary to reproduce its results; BASE along with the relative tutorials are hosted on GitHub: https://github.com/for‐giobbe/BASE.

## Data Availability

BASE along with the relative tutorials are hosted on GitHub: https://github.com/for‐giobbe/BASE.
